# Taxonomy, distribution, and natural history of flying foxes (Chiroptera, Pteropodidae) in the Mortlock Islands and Chuuk State, Caroline Islands

**DOI:** 10.3897/zookeys.345.5840

**Published:** 2013-10-29

**Authors:** Donald W. Buden, Kristofer M. Helgen, Gary J. Wiles

**Affiliations:** 1 Division of Natural Sciences and Mathematics, College of Micronesia–FSM, P.O. Box 159, Kolonia, Pohnpei 96941, Federated States of Micronesia; 2 Division of Mammals, National Museum of Natural History, Smithsonian Institution, Washington, D.C. 20013-7012, USA; 3 Washington Department of Fish and Wildlife, 600 Capitol Way North, Olympia, Washington 98501-1091, USA

**Keywords:** *Pteropus phaeocephalus*, *P. pelagicus*, *P. insularis*, *P. tokudae*, Mortlock Islands, Chuuk, Micronesia, atoll, taxonomy, distribution, status, natural history, climate change, sea level rise

## Abstract

The taxonomy, biology, and population status of flying foxes (*Pteropus* spp.) remain little investigated in the Caroline Islands, Micronesia, where multiple endemic taxa occur. Our study evaluated the taxonomic relationships between the flying foxes of the Mortlock Islands (a subgroup of the Carolines) and two closely related taxa from elsewhere in the region, and involved the first ever field study of the Mortlock population. Through a review of historical literature, the name *Pteropus pelagicus* Kittlitz, 1836 is resurrected to replace the prevailing but younger name *Pteropus phaeocephalus* Thomas, 1882 for the flying fox of the Mortlocks. On the basis of cranial and external morphological comparisons, *Pteropus pelagicus* is united taxonomically with *Pteropus insularis* “Hombron and Jacquinot, 1842” (with authority herein emended to Jacquinot and Pucheran 1853), and the two formerly monotypic species are now treated as subspecies — *Pteropus pelagicus pelagicus* in the Mortlocks, and *Pteropus phaeocephalus insularis* on the islands of Chuuk Lagoon and Namonuito Atoll. The closest relative of *Pteropus pelagicus* is *Pteropus tokudae* Tate, 1934, of Guam, which is best regarded as a distinct species. *Pteropus pelagicus pelagicus* is the only known resident bat in the Mortlock Islands, a chain of more than 100 atoll islands with a total land area of <12 km^2^. Based on field observations in 2004, we estimated a population size of 925–1,200 bats, most of which occurred on Satawan and Lukunor Atolls, the two largest and southernmost atolls in the chain. Bats were absent on Nama Island and possibly extirpated from Losap Atoll in the northern Mortlocks. Resident Mortlockese indicated bats were more common in the past, but that the population generally has remained stable in recent years. Most *Pteropus phaeocephalus pelagicus* roosted alone or in groups of 5–10 bats; a roost of 27 was the largest noted. Diet is comprised of at least eight plant species, with breadfruit (*Artocarpus* spp.) being a preferred food. Records of females with young (April, July) and pregnant females (July) suggest an extended breeding season. *Pteropus pelagicus pelagicus* appears most threatened by the prospect of sea level rise associated with global climate change, which has the potential to submerge or reduce the size of atolls in the Mortlocks. Occasional severe typhoons probably temporarily reduce populations on heavily damaged atolls, but hunting and ongoing habitat loss are not current problems for the subspecies.

## Introduction

Many islands of the west-central Pacific Ocean remain poorly known biologically, particularly the numerous, small, low-lying, coralline atolls and atoll-like islands of Micronesia. Their inaccessibility and relatively depauperate biotas (compared with those of larger and higher islands) have contributed to a paucity of visiting biologists. However, an understanding of the biogeography and biodiversity of Oceania remains incomplete without knowledge of the species that inhabit these miniscule lands.

The genus *Pteropus* is comprised of about 65 species of flying foxes, making it by far the largest genus in the family Pteropodidae (Simmons 2005; Helgen et al. 2009). Primarily island-dwelling, the genus is widespread through the Indo-Pacific region westward to the islands off eastern Africa. Much taxonomic work is still needed in the genus. Additionally, many species of *Pteropus* remain poorly known in terms of their population status, biology, and specific conservation needs. In particular, few studies of atoll-dwelling populations have been conducted (Dolbeer et al. 1988, Wiles et al. 1991, Holmes et al. 1994). The need for such studies is especially pressing in Pacific archipelagos, where larger bats have suffered considerable declines, extirpations, and extinctions over the past 200 years (Flannery 1995; Wiles et al. 1997; Helgen 2005; Helgen et al. 2009; Wiles and Brooke 2009, Nakamoto et al. 2012).

Although *Pteropus phaeocephalus* Thomas, 1882 was originally described as a distinct species endemic to the Mortlock Islands, a group of atolls in central Micronesia, there has long been recognition of the similarity between this form and *Pteropus insularis*, which is restricted to the neighboring main islands and barrier reef islands of Chuuk Lagoon and Namonuito Atoll located 171 km to the northwest (Oustalet 1895, Andersen 1912, Rainey and Pierson 1992, Kepler 1994, Flannery 1995). This has resulted in suggestions that *Pteropus phaeocephalus* may be better regarded as a subspecies or synonym of *Pteropus insularis* (Oustalet 1895; Koopman 1993; K. Helgen, in Simmons 2005). Information on the distribution, relative abundance, and ecology of *Pteropus phaeocephalus* is almost nonexistent, and the lack of comparative material from these islands, hitherto limited to the holotype, has impeded taxonomic appraisal. Specimens obtained from Namoluk and Satawan Atolls during this study provide material for new comparisons and a taxonomic reappraisal. Our field observations together with information provided by Mortlockese islanders furnish new data on the distribution, abundance, and biology of flying foxes in the Mortlocks. In light of the many threats to biodiversity on Pacific islands (Helgen et al. 2009, Wiles and Brooke 2009, Woinarski 2010), we also discuss conservation concerns relating to these bats.

## Study area

**Figure 1.**
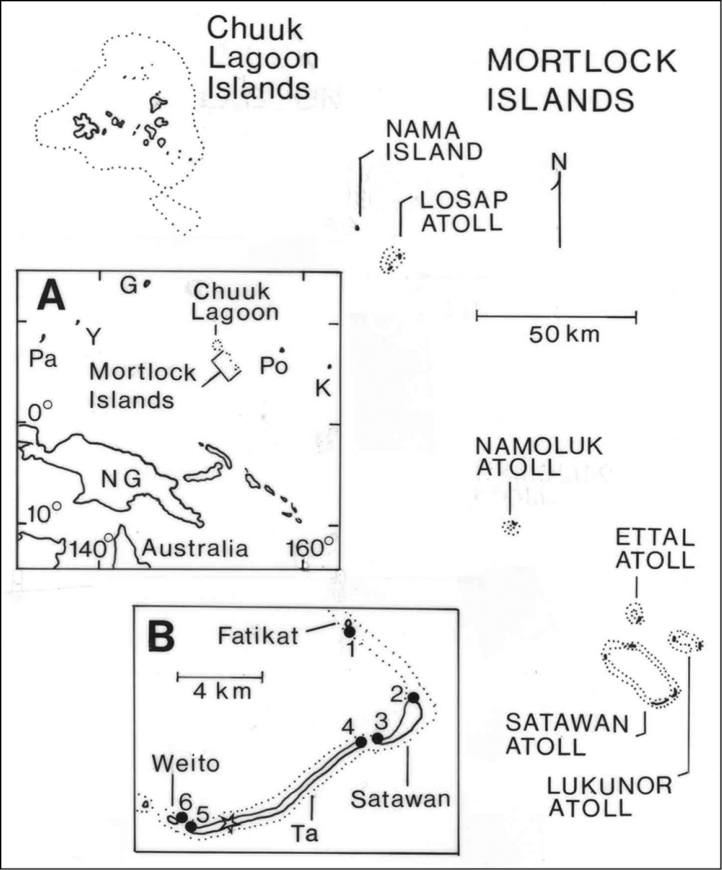
Location map for the Mortlock Islands and Chuuk Lagoon, Micronesia. Inset **A** location of islands in the west-central Pacific Ocean, G = Guam, K = Kosrae, NG = New Guinea, Pa = Palau, Po = Pohnpei, Y = Yap; inset **B** southern end of Satawan Atoll, solid circles indicate beach sites where interisland movement of flying foxes was assessed (see Table 5) and the open star indicates the airport station count site on Ta Island.

The Mortlock Islands (07°00'N, 152°35'E to 05°17'N, 153°39'E) are a part of Chuuk (formerly Truk) State, one of four states comprising the Federated States of Micronesia (FSM), the others being Yap, Pohnpei, and Kosrae. The FSM together with the Republic of Belau (Palau) make up the Caroline Islands in the tropical western Pacific Ocean. The Mortlocks are a chain of five atolls and one low, coral island spanning 224 km (Figure 1). Land area totals 11.9 km^2^, distributed among more than 100 islands; Ta, Satawan Atoll, is the largest island (Table 1). Maximum elevations are only 3–5 m asl. Over the years, a confusing array of alternative names and spellings has been proposed for different islands and island groups in the Mortlocks and Chuuk Lagoon (Baker 1951, Bryan 1971, Berg 1993). We use the names Northern Mortlocks for Nama Island and Losap Atoll, Central Mortlocks for Namoluk Atoll, and Southern Mortlocks for Ettal, Lukunor, and Satawan Atolls.

**Table 1.** Statistical data for the Mortlock Islands, Chuuk State, Federated States of Micronesia.

**Island group**	**Land area (km^2^)^a^**	**Number of islands**	**Largest island (km^2^)^a^**	**Number of inhabited islands**	**Number of residents^b^**	**Distance to next atoll (km)^c^**	**Observation days^d^**
**Northern Mortlocks**							
Nama Island	0.75	1	Nama (0.75)	1	995	14	7
Losap Atoll	1.03	10	Lewel (0.56)	2	875	110	1
**Central Mortlocks**							
Namoluk Atoll	0.83	5	Namoluk (0.31)	1	407	53	11
**Southern Mortlocks**							
Ettal Atoll	1.89	20^e^	Ettal (0.97)	1	267	7	3
Satawan Atoll	4.59	65^f^	Ta (1.55)	4	2,935	8	70
Lukunor Atoll	2.82	18	Lekinioch^g^ (1.28)	2	1,432	-	26

^a^ From Bryan (1971). ^b^ Based on the 2000 national census (Division of Statistics 2002). ^c^ Measured reef to reef. ^d^ Total number of days spent on the island(s) by DWB while conducting faunal surveys for flying foxes, birds, lizards, butterflies, and dragonflies. ^e^ Number of islands counted by DWB while walking on the reef flat, but Bryan (1971) recorded 18. ^f^ Based on information given DWB by residents of Satawan Atoll, but exact number uncertain. Bryan (1971) indicated “approximately” 49 islands in the summary section for “Truk District,” but mentioned at least 80 named and unnamed islands in the atoll and described one area in the northeast part of the atoll as having “numerous small cays on edge of reef” without naming or numbering them. ^g^ Formerly Lukunor Island, and known also as Likinioch, Lukinoch, and Lukunoch Island.

The Mortlocks fall within the equatorial rainbelt and are wet enough to support mesophytic vegetation (Mueller-Dombois and Fosberg 1998), though some of the smaller islets that lack a fresh water lens are more xeric. Coconut (*Cocos nucifera* L.) forest is the predominant vegetation type, with breadfruit (*Artocarpus* spp.) being a codominant tree in the interior of the larger islands. Other common forest and forest edge trees include *Barringtonia asiatica* (L.) Kurz, *Ficus* spp., *Guettarda speciosa* L., *Hernandia sonora* L., *Neisosperma oppositifolia* (Lam.) Fosb. & Sachet, *Pandanus* spp., and *Terminalia samoensis* Rech. Forest canopy height ranges from 10 to 20 m. The forest abuts the beach or merges abruptly with a narrow zone of coastal scrub or thicket dominated by *Tournefortia argentea* L. f. and *Scaevola taccada* (Gaertn.). Large community-maintained taro patches occupy much of the interior of Nama, Namoluk, Satawan, Kuttu, Moch, Oneop, and Lekinioch Islands. More detailed accounts of island vegetation and physiognomy exist for Losap Atoll (Severance 1976, Manner and Sana 1995), Namoluk Atoll (Marshall 1975), Ettal Atoll (Nason 1970), Lukunor Atoll (Borthwick 1977, Tolerton and Rauch n. d. [1949?]), and Kuttu Island, Satawan Atoll (Reafsnyder 1984).

All six island groups comprising the Mortlocks are inhabited, but only 1–4 islands in each group have permanent settlements (Table 1). The other islands are visited by atoll residents with varying degrees of frequency to cultivate taro or to gather coconuts, crabs, and other forest and coastal commodities. A total of 6,911 people lived in the Mortlocks in 2000, representing a density of 581 people per km^2^.

## Methods

Museum specimens utilized in this study are deposited in the collections of the American Museum of Natural History, New York, USA (AMNH), the Academy of Natural Sciences, Drexel University, Philadelphia, USA (ANSP), the Natural History Museum, London, UK (BMNH), Brigham Young University-Hawaii Campus, Laie, Hawaii, USA (BYUH), College of Micronesia-FSM, Kolonia, Pohnpei, Federated States of Micronesia (COM), the Museum of Comparative Zoology, Harvard University, Cambridge, Massachusetts, USA (MCZ), the Muséum National d’Histoire Naturelle, Paris, France (MNHN), the National Museum of Natural History, Smithsonian Institution, Washington, D.C., USA (USNM), and the Museum für Naturkunde, Humboldt University, Berlin, Germany (ZMB).

**Figure 2.**
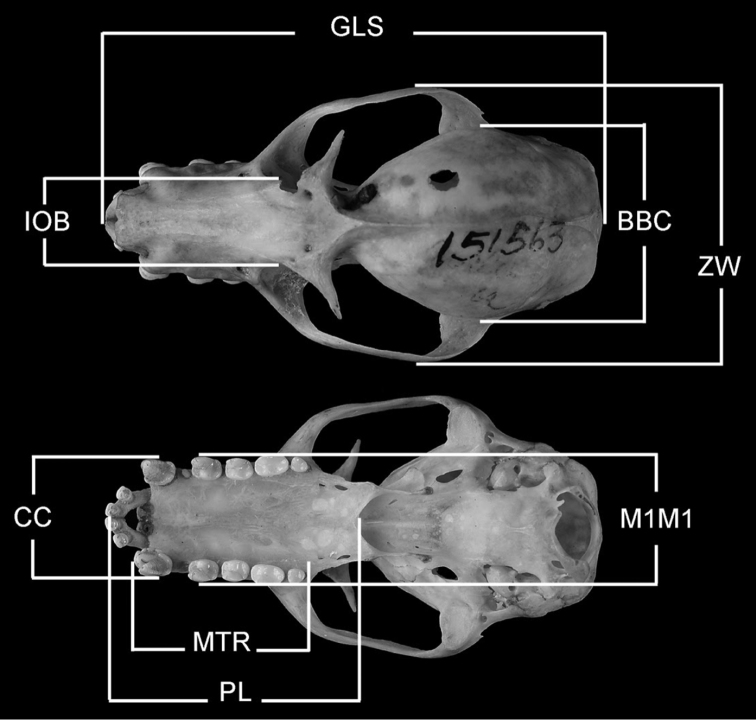
Cranial measurements employed in this study. See Methods for abbreviations.

Measurements of specimens are in millimeters and grams. Cranial and forearm measurements were taken on museum specimens (skins, skulls, and fluid preserved specimens) using dial calipers. Cranial measurements (see Figure 2) are abbreviated (and defined) as follows: GLS (greatest length of skull; distance from posterior midpoint on occipital crest to anterior midpoint of premaxillae); PL (palate length; distance from anterior midpoint of premaxillae to posterior midpoint of bony palate); ZW (greatest width across the zygomatic arches); IOB (width of interorbital constriction); BBC (breadth of braincase, measured across the braincase at squamosal bases); MTR (length of maxillary toothrow, alveolar distance from anterior root of canine to posterior root of last molar); CC (external, alveolar distance across upper canines); M1M1 (external, alveolar distance across upper first upper molars). Head-body length (snout to rump) and ear length were measured with a millimeter ruler on fresh material or recorded from museum specimen labels. Body mass was obtained from fresh specimens using Pesola scales or recorded from specimen labels. Immatures are defined as neonates to young weighing ≤100 g. Subadults are defined as adult-sized or nearly adult-sized young with incompletely fused skull sutures/synchondroses (cf. Helgen 2004) and some indication of cartilaginous epiphyseal swellings in the bones of the wing. Because sexual dimorphism is negligible in these bats, measurements for both sexes are pooled in our comparisons. Statistical methods included a two-sample *t*-test processed by Excel 2000 data analysis.

Standard descriptive statistics (mean, standard deviation, and observed range) were calculated for the samples of populations and species listed in the tables. Only cranial and dental measurements were incorporated in the multivariate analyses. Principal component analyses were computed using the combination of cranial and dental measurements indicated in tables and in the text. All measurement values were transformed to natural logarithms prior to multivariate analysis. Principal components were extracted from a covariance matrix. Variables for multivariate analyses were selected judiciously to maximize sample sizes for comparison by allowing for inclusion of partially broken skulls in some cases. The software program Statistica 8.0 (Statsoft Inc., Tulsa, Oklahoma, USA) was used for multivariate analysis.

Field work for this study began as part of a biological survey of terrestrial vertebrates and selected insect groups on Satawan Atoll, but was expanded in 2004 to include broader population assessments of flying foxes and other wildlife in the Mortlocks. Field surveys were conducted on Satawan Atoll during 17–26 December 2002, 7 July–1 August 2003, 30 March–9 April 2004, 22 June–6 July 2004, and 1–5 August 2004. The five other groups of islands were visited in 2004: Nama Island, 7–14 July; Losap Atoll, 10 July; Namoluk Atoll, 19–29 July; Ettal Atoll, 30 July–1 August; and Lukunor Atoll (Lekinioch Island only), 2–3 August. In 2012, incidental observations were made at Lukunor Atoll, 20 June–15 July, and Satawan Atoll, 15–17 July. All field work was conducted by DWB.

Information on the abundance and biology of bats came from a combination of sources. Station counts of flying bats were conducted on Satawan Atoll at two types of locations providing relatively unobstructed views of the sky: the Ta airport and six beach sites where interisland movement of bats was assessed (Figure 1). These counts were made by a single person at sunrise or sunset, lasted 25–95 minutes, and were made once or twice per time period at each station except for multiple counts at the Ta airport.

Flying foxes (and other wildlife) were also counted during walks conducted from mid-morning to mid- to late afternoon. These were made along pre-existing trails or by following a compass bearing through the center of less frequently visited islands, usually along the long axis of an island, but occasionally at right angles to it. Each route was surveyed only once. Care was taken to avoid double counting bats flushed by the observer. An undetermined, but probably substantial, proportion of roosting bats was hidden from view by the relatively dense forest canopy. Roosting flying foxes were viewed with 10x binoculars to observe behavior and search for the presence of young. Other information on bats was obtained from incidental observations and atoll residents. We did not search for feeding evidence of bats (i.e., discarded fruit, chewed pellets, fecal splats). An estimate of the number of flying foxes on each atoll was made based on: 1) overall numbers of bats seen, 2) percentage of each atoll covered by the observer, 3) the quality and amount of forest on an atoll, and 4) information provided by island residents, especially for Namoluk, Ettal, and Lukunor Atolls.

## Results

### Taxonomy. *Pteropus pelagicus* Kittlitz, 1836. Taxonomic history

The Russian research vessel *Senyavin*, under command of Friedrich [Feodor] Lütke, and with F. H. von Kittlitz as one of the ship’s naturalists, was in the eastern Caroline Islands early in 1828 (Nozikov 1946). After leaving Ualan (= Kosrae), the *Senyavin* spent approximately the first two weeks of February in the southern Mortlocks (Lütke 1835). Although the ship traveled along the coasts of all three southern atolls, the only anchorage and the base of scientific operations was the harbor at Lukunor Atoll (Lütke 1835). Remarking on the fauna of Lukunor, Kittlitz (1836) stated “Nous prîme, comme à Ualan, deux mammifères dont l’un est un *Pteropus* (*Pteropus pelagicus* m.) qui sans aucun doute diffère de celui de cette île, tant par sa taille qui est beaucoup moindre, que par une tache blanche de forme circulaire qu’il a sur l’abdomen [We found, as on Kosrae, two mammals, one of which is a *Pteropus* (*Pteropus pelagicus* mihi) that without any doubt differs from the one of that island by its much smaller size and by a round white patch on its abdomen].” Although Kittlitz’s description is brief and unaccompanied by illustration, clearly he was referring to the same species that Thomas (1882: 756) named *Pteropus phaeocephalus*. The zoological specimens that Kittlitz obtained during this expedition were deposited in the Russian Academy of Sciences in St. Petersburg (Baker 1951, Hume 2001), but the only flying foxes in the St. Petersburg collection today are two *Pteropus pselaphon* Say from the Bonin Islands, Japan (V. Loskot, pers. comm.). We thus regard *Pteropus pelagicus* as the earliest name for the flying fox currently known as *Pteropus phaeocephalus*, and advocate its usage (see Taxonomic Conclusions, below).

Thomas (1882) described *Pteropus phaeocephalus* from the Mortlock Islands based on “a gravid female in alcohol” collected by Dr. [John = Johann = Jan] Kubary for the Godeffroy Museum, Hamburg, Germany, and subsequently transferred to the Natural History Museum, London (BMNH 82.10.27.4). Andersen (1912: 299) considered the holotype as “perfectly similar to *Pteropus insularis* [from Chuuk Lagoon islands] in skull, dentition, palate-ridges, and all external characters, except much paler colour of the fur, particularly on the back.” He (Andersen 1912: 299) concluded “the evidence is in favour of the assumption that *Pteropus phaeocephalus* (Mortlock group) is specifically distinct from *Pteropus insularis* (Ruck [= Chuuk] group).” This opinion has prevailed in the literature until relatively recently. Koopman (1993) remarked that *Pteropus phaeocephalus* is probably better recognized as a subspecies of *Pteropus insularis*, and Simmons (2005) concurred in referring to the Mortlock population as *Pteropus insularis phaeocephalus*.

The holotype of *Pteropus phaeocephalus* is labeled as collected by Kubary in the Mortlock Islands, but the date and the name of the island are unstated. Kubary traveled widely in the Pacific while employed as a naturalist and ethnographer by the Godeffroy Museum (Spoehr 1963). He visited the Mortlock Islands apparently on two occasions. Hezel (1979: 33, revised online as of 2002 and unpaged) stated that the brig *Iserbrook* put in at Ta, Satawan Atoll on 16 February 1873 and “returned in July or August with John Kubary aboard from Palau.” Referring to the same voyage, Paszkowski (1971: 48) stated that “in May, 1873, Kubary sailed on the *Iserbrook* [from Palau] and visited the islands of Ngulu, Ulithi, Woleai, Nukuoro, and Mortlock…[and] landed at Ponape [= Pohnpei] in August, 1873.” Little is known of Kubary’s activities in the Mortlocks during this visit, which may have been very brief, and Kubary did not allude to it in his ethnography of the Mortlocks (Kubary 1880). Also, any specimens that may have been collected at this time may have been among those lost in a subsequent shipwreck at Jaluit, in the Marshall Islands, in September 1874, when only a small part of Kubary’s collections of the previous five years were saved (Paszkowski 1971, 1987).

Kubary returned to the Mortlocks in 1877. In a separate and anonymously written introduction to the ethnography that Kubary apparently sent to L. Friederichsen, editor of Mittheilungen der Geographischen Gesellschaft in Hamburg, the author (L. Friederichsen?) or authors (in Kubary 1880) stated “Kubary verweilte auf den Mortlock-Inseln während der Monate März bis Ende Mai 1877 und zwar speciell auf der Inseln Tä, Uoytä und Aliar [Kubary visited the Mortlock Islands, particularly the islands Ta, Weito, and Aliar during the months of March through the end of May 1877].” Westwood (1905: 89, 112) saw Kubary also on Satawan Island (immediately to the east of Ta) during his visit in 1877. In light of this information, the holotype of *Pteropus phaeocephalus* probably was collected on Satawan Atoll sometime during March–May 1877 and very likely on Ta Island, where flying foxes are now common and where Kubary seems to have spent most of his time during his three months in the Mortlocks (Nason 1970: 363).

Matschie (1899) discussed a specimen he referred to as *Pteropus phaeocephalus* from Pohnpei in the Caroline Islands, obtained by Otto Finsch (ZMB A4065), but this is actually a specimen of a *Pteropus* allied to *Pteropus mariannus* (Andersen 1912), previously discussed as a specimen of *Pteropus ualanus*, otherwise known only from Kosrae (Wiles et al. 2008) but in fact most closely resembling *Pteropus pelewensis* of Palau (confirmed by KMH). The source of Finsch’s specimen was possibly mislabeled or the specimen may represent a population of *Pteropus pelewensis* (or a closely related taxon) that has become extinct on Pohnpei, where only *Pteropus molossinus* occurs today (Flannery 1995).

### Morphological comparisons. External comparisons

**Figure 3.**
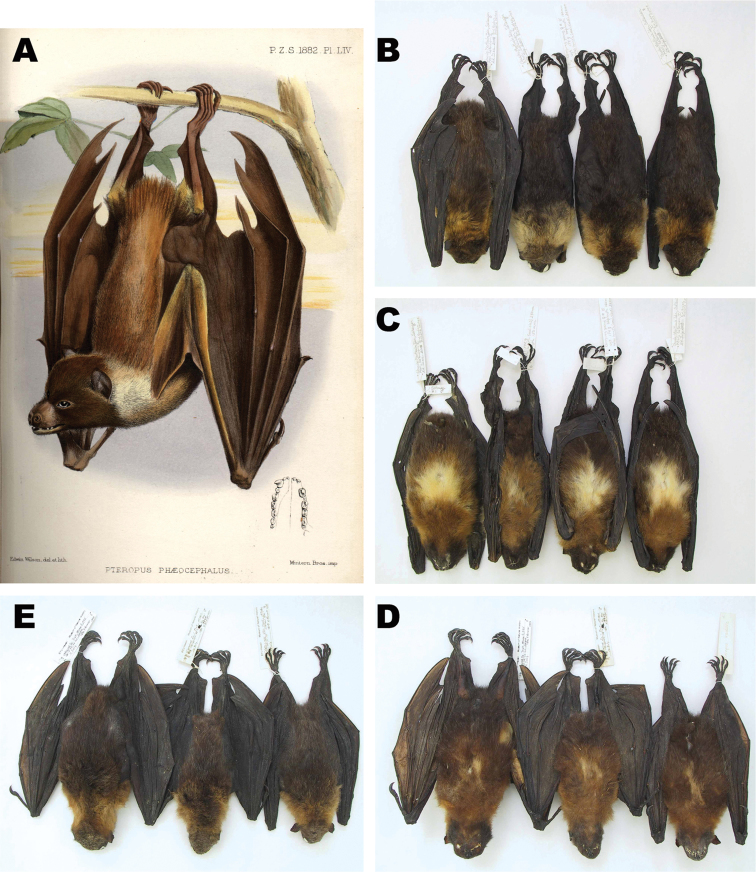
(**A**) Scanned image of Plate LIV from original description of *Pteropus phaeocephalus* Thomas 1882, which is presumably based on the holotype (**B**) dorsal view and (**C**) ventral view of *Pteropus pelagicus pelagicus* from Satawan Atoll (**D**) ventral view and (**E**) dorsal view of *Pteropus phaeocephalus insularis* from Weno, Chuuk Lagoon.

Among the 11 specimens from Satawan Atoll, the mantle ranges from creamy white to deep buff or tawny in adults and subadults, and is grayish brown (without yellowish or reddish highlights) in immatures. It averages darker (more tawny, less pale buff) in the six adults from Namoluk Atoll. In all Mortlock specimens, the mantle is bisected by a narrow to broad area of darker (brown) pigmentation, which, in some cases, gives the appearance of having pale shoulder patches. In specimens from both Namoluk and Satawan, the back and rump are dark brown (occasionally tinged with reddish brown in Satawan specimens), with scattered pale-tipped hairs; Namoluk specimens average slightly darker than those from Satawan. In both the Namoluk and Satawan material, the face is dark brown to nearly black and with varying amounts of grizzling. The top of the head is brown or grayish brown. The venter is usually light brown to pale reddish brown anteriorly (on throat and chest) and dark brown posteriorly; subadults are more uniformly light brown. A large prominent white or pale yellowish white patch, 20–40 mm wide, occurs midventrally in most specimens (Figure 3C); it is occasionally slightly reduced or nearly obsolete.

Mortlock Islands specimens generally match the color descriptions of the holotype of *phaeocephalus* as described by Thomas (1882) and Andersen (1912), although the pale-tipped hairs on the dorsum described as “nearly white” by Thomas (1882) and “golden cream buff” by Andersen (1912) are much sparser in the new material and visible only upon close inspection. Mortlock specimens, especially some of the paler and brighter individuals from Satawan, closely resemble the illustration (Plate LIV) in Thomas (1882) (see Figure 3A). DWB observed an unusually pale individual through binoculars from a distance of 30–40 m on Toimon Island, Namoluk Atoll, on 27 July 2004. The venter and sides were entirely creamy white. Only the top of the head, neck, a small part of the mantle middorsally, and most of the back and rump were dark brown.

Compared with a sample of five specimens of *Pteropus insularis*, all presumably from Chuuk Lagoon (four from Weno, one [MCZ 7023] of uncertain origin), the Mortlock Islands bats tend to show greater contrast between the paler coloration of the mantle and the darker pigmentation on the back and rump. The midventral pale patch is consistently larger in the Mortlock specimens that we examined, although Andersen (1912: 296) stated “the size of this bright pectoral patch varies greatly individually [in *Pteropus insularis* from Chuuk Lagoon], but in none is it wanting….” The cotype of *Pteropus insularis* from Chuuk Lagoon illustrated in Hombron and Jacquinot (1844: plate v) shows a prominent mid-ventral patch as large as any seen in specimens from the Mortlocks (George Glazer Gallery web page at www.georgeglazer.com/archives/prints/animals/durvillebat.html ). Hair length was measured only on the mantle and averaged slightly shorter in Chuuk Lagoon specimens than in those from the Mortlocks—14.7 mm long in three specimens from Weno, Chuuk Lagoon, and 15.0 and 16.0 mm in four from Namoluk and nine from Satawan, respectively. In body size, bats from Chuuk Lagoon are similar to those from the Mortlocks. One skin and skull (AMNH 249954) dated 26 May 1987 and 13 other skulls (AMNH 249955–67) dated 3 July 1986 were shipped from Chuuk State and intercepted by customs officials in Guam; the specimens were prepared by G. Wiles. Whether the AMNH series consists entirely of specimens from Chuuk Lagoon, which is likely, or whether it includes material from the Mortlocks or other Chuuk outliers, is unknown. During the 1980s, bats commercially hunted in the Mortlocks were transshipped to Weno for transfer to Guam and the Commonwealth of the Northern Mariana Islands together (but apparently unsegregated) with bats collected on islands in Chuuk Lagoon. In external measurements, the AMNH specimens are similar to samples from both Weno and those from the Mortlocks (Table 2). Chromatically, the lone AMNH skin more closely resembles those from Satawan in the Mortlocks than it does the small sample from Weno.

**Table 2.** Mensural data for samples of flying foxes from Chuuk Lagoon islands and the Mortlock Islands; data sets include range, *n* in parentheses, and mean ± SD; all measurements are in millimeters or grams.

				**Mortlock Islands**	
**Character**	**Sex**	**“Truk”^a^**	**Chuuk Lagoon islands**	**Namoluk Atoll**	**Satawan Atoll**
Head-body length	*♂*	____	168.0–186.0 (2) 177.0 ± 12.73	158.0 (1)	150.0–170.0 (2) 160.0 ± 14.0
	*♀*	165.0 (1)	131.0 (1)	155.0–170.0 (5) 163.0 ± 6.08	155.0–170.0 (4) 161.3 ± 6.29
Forearm length	*♂*	____	101.0 (1)	103.9 (1)	102.0–108.7 (2) 105.4 ± 4.74
	*♀*	104.8 (1)	____	102.3–107.3 (5) 104.6 ± 2.18	103.2–108.0 (5) 105.3 ± 1.78
Ear length	*♂*		20.0–22.0 (2) 21.0 ± 1.41	23.0 (1)	24.0 (1)
	*♀*	____	____	23.0–24.0 (3) 23.7 ± 0.58	22.0–23.0 (2) 22.5 ± 4.74
Body mass	*♂*	____	148.0–200.0 (2) 174.0 ± 36.76	175.0 (1)	142.0–203.0 (2) 172.5 ± 43.1
	*♀*	170.0 (1)	150.0 (1)	190.0–225.0 (5) 211.0 ± 13.87	153.0–187.0 (4) 171.8 ± 12.28
Greatest skull length (GLS)	*♂*	43.9–46.1 (5) 44.8 ± 0.18	44.6 (1)	45.0 (1)	47.2 (1)
	*♀*	44.0–45.4 (5) 44.7 ± 0.53	44.1 (1)	45.3–46.1 (2) 45.7 ± 0.57	45.0–46.6 (4) 45.7 ± 0.59
Palate length (PL)	*♂*	22.6–24.0 (5) 23.0 ± 0.59	22.2 (1)	23.3 (1)	22.0–24.4 (2) 23.2 ± 1.70
	*♀*	22.0–24.0 (6) 22.8 ± 0.75	22.9 (1)	23.0–24.0 (2) 23.5 ± 0.71	23.2–24.1 (3) 23.9 ± 0.59
Maxillary toothrow (MTR)	*♂*	14.8–15.2 (5) 15.1 ± 0.17	15.1–15.2 (2) 15.2 ± 0.70	15.5 (1)	15.2–15.8 (2) 15.6 ± 0.50
	*♀*	15.0–15.5 (6) 15.2 ± 0.19	14.8 (1)	14.8–15.5 (3) 15.2 ± 0.36	15.0–15.5 (4) 15.2 ± 0.24
Breadth of braincase (BBC)	*♂*	17.2–17.6 (5) 17.4 ± 0.15	17.3 (2)	18.0 (1)	17.6–18.2 (2) 17.9 ± 0.42
	*♀*	17.0–17.8 (6) 17.5 ± 0.33	17.0 (1)	17.9–18.1 (2) 18.0 ± 0.14	17.5–18.1 (4) 17.9 ± 0.26
Breadth across canines (CC)	*♂*	10.0–11.0 (5) 10.4 ± 0.44	10.8–11.1 (2) 11.0 ± 0.21	11.1 (1)	10.7–11.9 (2) 11.3 ± 0.85
	*♀*	9.6–10.8 (6) 10.4 ± 0.41	9.9 (1)	11.0–11.4 (3) 11.2 ± 0.21	10.9–11.1 (4) 11.0 ± 0.10
Breadth across M^1^ (M1M1)	*♂*	12.0–12.5 (4) 12.3 ± 0.22	12.2 (2)	12.0 (1)	12.3–12.9 (2) 12.6 ± 0.42
	*♀*	12.1–12.8 (5) 12.3 ± 0.29	12.0 (1)	11.9–12.3 (3) 12.1 ± 0.21	12.3–12.8 (3) 12.6 ± 0.26)
Interorbital breadth (IOB)	*♂*	7.0–7.4 (4) 7.2 ± 0.17	7.5 (1)	7.5 (1)	7.1–7.6 (2) 7.4 ± 0.35
	*♀*	7.0–7.4 (6) 7.2 ± 0.14	7.2 (1)	7.7–8.0 (3) 7.8 ± 0.15	7.7–7.8 (3) 7.8 ± 0.06
Zygomatic width (ZW)	*♂*	23.0–25.4 (4) 24.1 ± 0.95	24.0–27.0 (2) 25.5 ± 2.21	25.6 (1)	23.9–27.1 (2) 25.5 ± 2.26
	*♀*	23.6–25.4 (5) 24.5 ± 0.65	____	25.7–26.0 (2) 25.9 ± 0.21	25.2–25.6 (3) 25.4 ± 0.20

^a^ Specimens confiscated on Guam, but originated from Chuuk State. Most were probably collected on islands in the Chuuk Lagoon, but some may have come from outer atolls, including the Mortlocks.

The type series of *Pteropus laniger*, originally described as *Pteropus lanigera* [sic]by H. Allen (1890), and now regarded as a synonym of *Pteropus phaeocephalus insularis* (Andersen 1912; Helgen et al. 2009) (USNM 37815, lectotype, and MCZ 7023, paralectotype), was stated by Allen (1890) to have come from Samoa, but this was later emended to the Caroline Islands (see Andersen 1912; Helgen and McFadden 2001). These specimens match well with BYUH specimens from Weno in being nearly uniformly brown with less contrasting areas of coloration, and having a reduced midventral pale patch, and probably originated from one of the main islands of Chuuk Lagoon.

**Figure 4.**
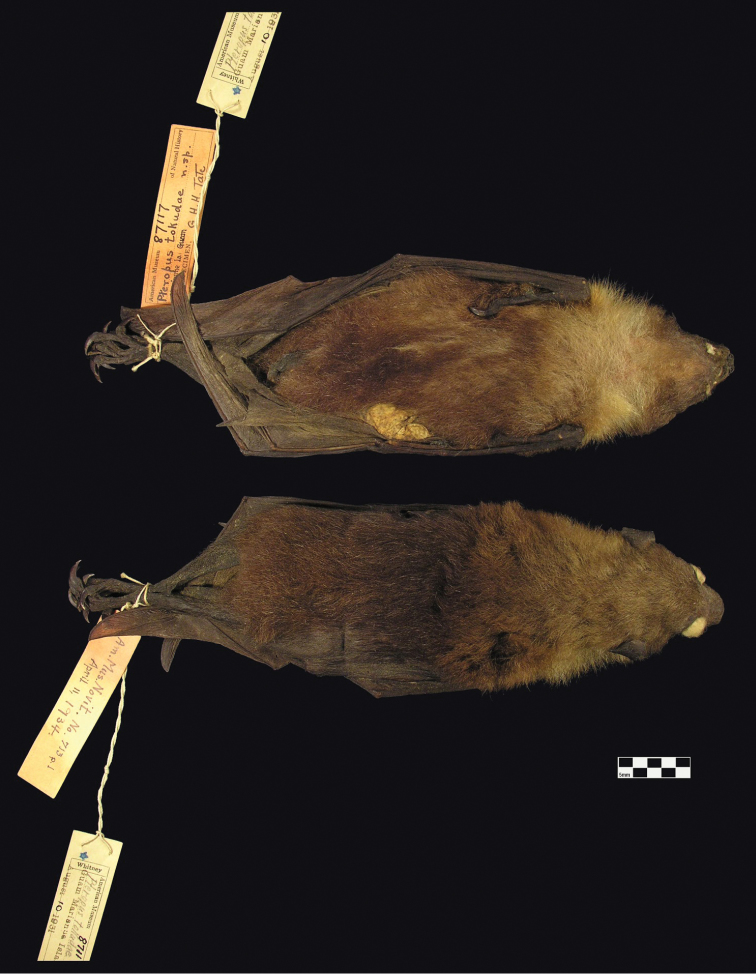
Holotype skin of *Pteropus tokudae*. AMNH 87117, adult male, from Guam, collected 10 Aug 1931 by W.F. Coultas. Scale bar = 25 mm.

Overall pelage coloration of the two available skins of *Pteropus tokudae* (e.g. Figure 4; see further discussion below) is generally similar to that of *Pteropus phaeocephalus insularis*, perhaps averaging somewhat darker overall (Tate 1934; Flannery and Schouten 2001: 172).

## Craniometric comparisons

Our study of essentially all available museum specimens of *Pteropus* from the islands of Chuuk Lagoon, Namonuito Atoll, and the Mortlocks, including the newly collected Mortlock material reported here, allowed us to examine patterns of multivariate craniometric variation in *Pteropus* from these archipelagos using Principal Component Analyses (PCA). We also included in these comparisons the three available skulls of *Pteropus tokudae* Tate, 1934, of Guam (a species that apparently became extinct in the late twentieth century: Wiles 1987a), which appears to be the closest relative of *Pteropus pelagicus*. *Pteropus tokudae* and *Pteropus pelagicus* share most qualitative morphological features but purportedly differ in that *Pteropus tokudae* is darker brown in coloration and smaller in body size (Tate 1934; Flannery 1995); Tate (1934) indicated in the original description of *tokudae* that, “It may well be merely a race of *insularis*.” We included *Pteropus tokudae* in these comparisons because no study has yet explored the consistency of differences between *Pteropus tokudae* and *Pteropus pelagicus*, and because, as noted above, the largest available sample of *Pteropus* specimens supposedly originating from Chuuk State was seized in Guam, such that we wanted to evaluate whether these specimens showed closer craniometric relationship with *Pteropus phaeocephalus insularis* of Chuuk Lagoon, or with *Pteropus tokudae* of Guam. Demonstration of the precise phylogenetic relationships of *Pteropus pelagicus* and *Pteropus tokudae* awaits a more detailed molecular phylogenetic review of the genus *Pteropus* than has yet been published (Giannini et al. 2008), but we regard *Pteropus pelagicus* and *Pteropus tokudae* to be closely related species that constitute a small radiation of relatively small-bodied flying-foxes from islands of the remote Central Pacific.

**Figure 5.**
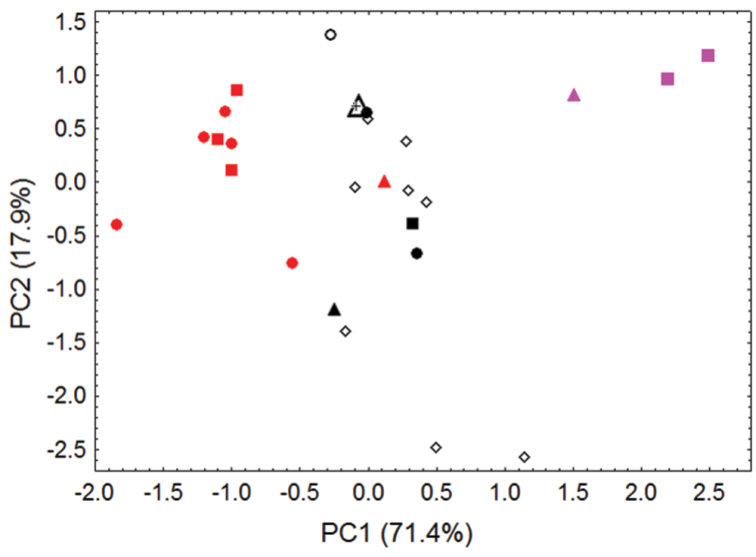
Morphometric separation (first two principal components of a Principal Components Analysis) of 27 adult skulls of *Pteropus pelagicus* and *Pteropus tokudae*. These comparisons involve 5 measurements (maxillary toothrow length, breadth of braincase, external breadth of rostrum across canines, external breadth of palate across first upper molars, and least interorbital breadth). The first principal component mainly reflects distinctions in overall skull size, which increases from right to left. Specimens of *Pteropus pelagicus pelagicus* from the Mortlock Islandsare denoted by red symbols (including the red triangle, the holotype of *phaeocephalus* from “Mortlock Islands”; red circles, Satawan Atoll; and red squares, Namoluk Atoll). Specimens of *Pteropus pelagicus insularis* are denoted by black symbols; closed black symbols indicate samples of known geographic origin (including the closed black triangle, the holotype of *insularis* from “Ruck”; closed black circles, specimens labeled “Ruck”; closed black square, specimen labeled “Uala” (= Weno); and black cross, specimen from Namonuito Atoll) and open symbols indicate specimens of imprecise geographic origin (including the large open triangle, the lectotype of *laniger*, erroneously attributed to the “Samoa Islands” in the original description; open black circle, an unprovenanced specimen from ANSP; and open black diamonds, specimens at AMNH seized on Guam but originating from Chuuk State). Specimens of the closely related species *Pteropus tokudae* from Guam are denoted by pink symbols (including the pink triangle, the holotype of *tokudae* from Guam, and the pink squares, other specimens from Guam).

The type of *pelagicus* can no longer be traced (see above), and the skulls of the surviving type specimens for the other relevant taxa in this group (*insularis* [MNHN 1996-2112, syntype of *insularis*, designated here as the lectotype of *insularis* based on being the only skull available from the type series], *phaeocephalus* [BMNH 82.10.27.4, holotype], *laniger* [USNM 37815, lectotype], and *tokudae* [AMNH 87117, holotype] are all somewhat broken. Consequently, we based our PCA comparisons on a relatively small number of five variables (Table 4, Figure 5) to allow for the inclusion of these various type specimens, as well as various other museum specimens (adult skulls at AMNH, ANSP, BMNH, COM, MNHN, USNM, and ZMB), many of which are also partially broken.

**Table 3.** Cranial measurements (mean ± SD, in millimeters, *n* in parentheses) for pooled samples of flying foxes from Chuuk Lagoon islands versus Mortlock Islands^a^.

**Character**	**Chuuk Lagoon^b^**	**Mortlock Islands**	***t***	**df**	**P**
Greatest skull length	44.8 ± 0.63 (12)	45.9 ± 0.80 (6)	-3.37	18	0.003
Palate length	22.8 ± 0.64 (13)	23.5 ± 0.82 (8)	-2.22	19	0.039
Maxillary toothrow	15.1 ± 0.18 (14)	15.3 ± 0.32 (10)	-1.93	22	0.066
Breadth of braincase	17.4 ± 0.26 (14)	17.9 ± 0.24 (9)	-4.88	21	0.000
Breadth across canines	10.5 ± 0.44 (14)	11.1 ± 0.34 (10)	-3.93	22	0.001
Breadth across M^1^	12.4 ± 0.30 (12)	12.3 ± 0.36 (9)	0.21	19	0.834
Interorbital breadth	7.2 ± 0.16 (12)	7.7 ± 0.26 (9)	-4.83	19	0.000
Zygomatic width	24.5 ± 1.07 (11)	25.6 ± 0.89 (8)	-2.21	17	0.041

^a^ Unpooled sample statistics are in Table 2. ^b^ Includes AMNH specimens confiscated on Guam.

Our craniometric comparisons (Figure 5, Table 4) indicate that skulls from the Mortlock Islands (*pelagicus*), from the islands of Chuuk Lagoon and Namonuito Atoll (*insularis*), and from Guam (*tokudae*), separate primarily along the first principal component (representing 71% of the variance), mainly reflecting differences in overall skull size between these insular populations/taxa.

**Table 4.** Factor loadings, eigenvalues, and percentage of variance explained by illustrated components (Figure 5) from Principal Components Analysis of 27 adult skulls of *Pteropus pelagicus* and *Pteropus tokudae* (see “specimens examined”). Principal components are extracted from acovariance matrix of 5 log-transformed cranialmeasurements (see Figures 2 and 5, Tables 2–3).

**Variable**	**PC1**	**PC2**
Interorbital width	-0.587	0.798
Breadth of braincase	-0.869	-0.121
Maxillary toothrow length	-0.911	-0.163
Breadth across canines	-0.972	-0.105
Breadth across M^1^s	-0.711	-0.368
Eigenvalue	0.013	0.003
Percent variance	71.4%	17.9%

**Figure 6.**
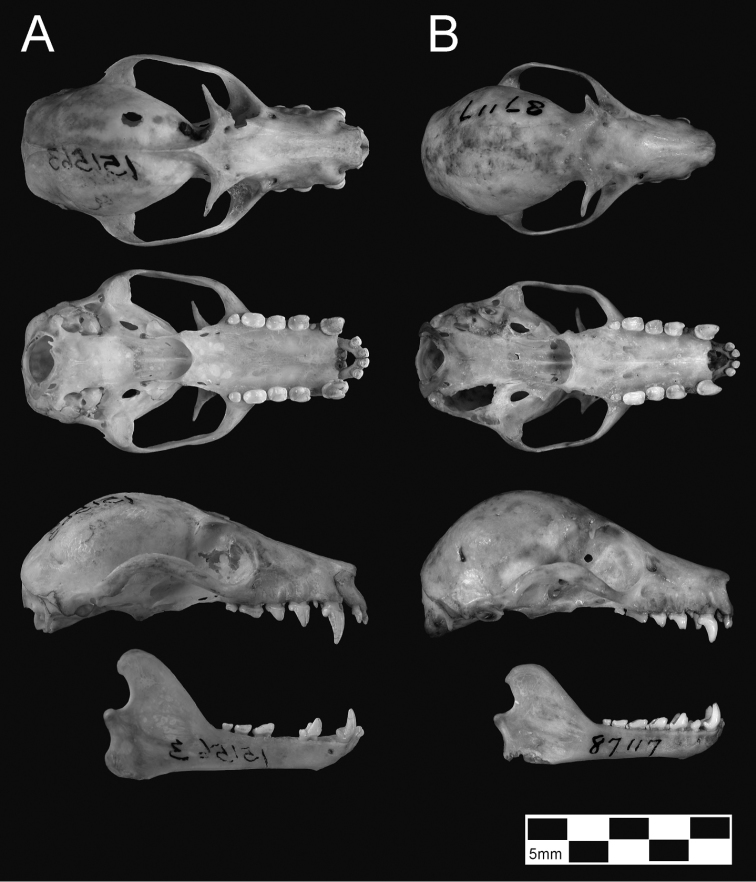
Skulls of *Pteropus pelagicus* (**A** *Pteropus phaeocephalus insularis*, USNM 151563, unsexed adult, from Uala [= Weno]; note last lower premolars missing in lower jaw) and *Pteropus tokudae* (**B** AMNH 87117, holotype of *tokudae*, adult male, from Guam). Scale bar = 25 mm.

Several points are of special note. First, specimens of *tokudae* separate cleanly from *pelagicus/insularis*, chiefly on the basis of their consistently smaller size (showing no overlap with *Pteropus pelagicus* in most skull measurements). *Pteropus tokudae* has a markedly smaller skull (greatest length 41.2–42.2 mm) with a relatively shorter and narrower rostrum compared to *Pteropus pelagicus* (Figure 6).

Second, some overlap in morphometric space characterizes samples of *pelagicus* (skulls generally larger)and *insularis* (skulls generally smaller), especially with regard to the holotype of *Pteropus phaeocephalus*, which associates more closely with Chuuk Lagoon and Namonuito specimens than with more recent Mortlock samples. Chuuk Lagoon and Namonuito specimens tend to average slightly smaller in most measurements than Mortlock specimens (Table 2), but sample sizes are small. Statistically significant differences (*p* < 0.05) were found in six of eight cranial measurements when samples from different localities were pooled and sexes combined (Tables 2–3); ranges overlap in all measurements.

The only available skull from Namonuito Atoll (BMNH 15.1.18.1) shows closest association with specimens from Chuuk Lagoon (Figure 5), an indication that the flying fox population of Namonuito, the taxonomic position of which has not previously been analyzed and has been considered uncertain (Rainey and Pierson 1992, Flannery 1995), is best referred to *Pteropus phaeocephalus insularis* (but its taxonomic status should ideally be re-evaluated once additional comparative material is available). The lectotype of *laniger* also associates craniometrically with specimens of *Pteropus phaeocephalus insularis* (Figure 5), supporting the suggestion that the type series of *laniger* originated from Chuuk Lagoon (Andersen 1912, Helgen and McFadden 2001, Helgen et al. 2009) and that *laniger* can be regarded as a synonym of *Pteropus phaeocephalus insularis*. The majority of specimens at AMNH that were seized on Guam and said to originate from Chuuk State fall within the range of morphometric variation for specimens known to have come from Chuuk Lagoon (Figure 5), supporting the idea that they likely originated from Chuuk Lagoon, and their taxonomic identification as *Pteropus phaeocephalus insularis*. However, the AMNH series, along with an unprovenanced specimen from the ANSP (ANSP 6196), expand the range of variation in *Pteropus phaeocephalus insularis* along the second principal component (representing 18% of the variance), suggesting a slightly greater range of cranial shape variation within *Pteropus phaeocephalus insularis* than documented by firmly localized samples. The possibility that some of these unprovenanced AMNH and ANSP specimens might have originated from the Mortlocks, or other outlying island populations of Chuuk State, cannot be ruled out.

Our search for museum material of *Pteropus phaeocephalus pelagicus* and *Pteropus phaeocephalus insularis* uncovered one additional very interesting and largely overlooked museum specimen. This is a broken skull of a flying fox much larger than *Pteropus phaeocephalus insularis*, received from Otto Finsch, probably in 1880 (when he was in the Caroline Islands during his first Pacific expedition), and labeled “Ruck” (= Chuuk). This specimen (ZMB 5697) was misidentified as *Pteropus insularis* by Matschie (1899), who figured the skull (Matschie 1899: Plate 5, numbers 11-12), but was correctly identified by Andersen (1912) as a member of the remote Pacific group of flying foxes allied taxonomically with *Pteropus mariannus* (including the named forms *pelewensis, yapensis*, and *ualanus*). This specimen, examined by KMH, most closely resembles *Pteropus pelewensis* of Palau and provides an indication that, like Guam (the original fauna of which included *Pteropus mariannus* and *Pteropus tokudae*), the islands of the Chuuk Lagoon may have supported both a smaller species of flying fox (*Pteropus phaeocephalus insularis*) and *Pteropus* cf. *pelewensis*. No other collections or reports from Chuuk State give any indication of a larger species of flying fox in the archipelago, suggesting that any larger taxon that may have inhabited these islands is now extinct. However, the possibility that it could still exist in an outlying island group in Chuuk State should be kept in mind by future fieldworkers in these regions. Similarly, and as noted above, Finsch’s overlooked nineteenth century Pacific collections in Berlin reveal that the fauna of Pohnpei also may have included both a small species, *Pteropus molossinus*, which survives, and a larger species, also most closely resembling *Pteropus pelewensis*, unknown today in Pohnpei. Alternatively, we cannot fully discount the possibility that the two anomalous specimens (one from Chuuk, the other from Pohnpei) are specimens of *Pteropus pelewensis* from Palau that have been mislabeled. Both were obtained by Finsch and deposited in the collections of the Berlin Museum (ZMB). Finsch visited Pohnpei in 1880 (Finsch 1881), but we found no mention of his having travelled to Chuuk or Palau. However, while on Pohnpei, he visited with Johann Kubary, who resided at Pohnpei at the time and who had collected zoological specimens extensively throughout the Caroline Islands, including in Chuuk and Palau. Finsch (1880) published many new locality records of birds from Chuuk State based on Kubary’s specimens that he (Finsch) examined during this visit. Possibly the enigmatic specimen of flying fox from “Ruck” originated from Kubary’s collections. The likelihood of mislabeling in the cases of these two specimens is further enhanced by the tendency of the curators at the Berlin Museum at that time to remove collectors’ labels when attaching uniformly standardized museum labels (Frahnert and Buden 2010).

### Taxonomic conclusions

The name *Pteropus pelagicus* Kittlitz, 1836 is a senior synonym of the younger but prevailing name *Pteropus phaeocephalus* Thomas, 1882. Kittlitz’s name is listed in Sherborn’s (1929) Index Animalium (Part XIX: 4818), which Gardner and Hayssen (2004) included in a list of important nomenclators in mammalogy. It is also used in a similar context in Kramer’s (1935) report on the results of the German 1908–1910 South Seas Expedition. Inasmuch as the name *Pteropus pelagicus* appears in at least these two post-1899 publications, it is not a “forgotten name” (*nomen oblitum*) *sensu* ICZN (1999: Article 23.9 and glossary), and we find no compelling reason to maintain use of the younger name under special provisions of the Code (ICZN 1999: Article 23.9.3). The species known largely by its junior synonym, *Pteropus phaeocephalus*, has a very limited distribution, has until now been known by a single museum specimen, and has been rarely discussed in the literature. In a strict application of the Principle of Priority, the name *Pteropus pelagicus* Kittlitz, with type locality Lukunor Atoll in the Mortlock Islands, is resurrected here to replace the younger synonym, *Pteropus phaeocephalus* Thomas.

In view of the very close similarity in body size and cranial features and measurements between specimens from Chuuk Lagoon islands (*Pteropus insulari*s) and those from the Mortlocks Islands (*Pteropus pelagicus*), we include *Pteropus insularis* Jacquinot and Pucheran, 1853 (herein emended from Hombron and Jacquinot, 1842 – see remarks on authorship, this account) in *Pteropus pelagicus* and treat the two former monotypic species as subspecies – *Pteropus phaeocephalus pelagicus* Kittlitz, 1836 in the Mortlock Islands (synonym *phaeocephalus* Thomas, 1882) and *Pteropus phaeocephalus insularis* Jacquinot & Pucheran, 1853 on Chuuk Lagoon islands and Namonuito Atoll (synonym *laniger* Allen, 1890). Nomenclatorial issues notwithstanding, this arrangement follows in principle that of Simmons (2005), who referred to the Mortlock population as *Pteropus insularis phaeocephalus*. The nominate subspecies (*Pteropus phaeocephalus pelagicus*) is distinguished from *Pteropus phaeocephalus insularis* chiefly, and on average, by its brighter coloration, more contrastingly colored (paler) mantle, larger white or pale buff midventral patch, and larger cranial size. The euphonious English vernacular name “Pelagic flying fox” would logically stem from the binomen, but we suggest the name “Chuuk flying fox” for this species following a tendency to use geographic-based names for the pteropodids endemic to the Caroline Islands (e.g. Flannery 1995, Wilson and Cole 2000, Simmons 2005, Wiles 2005).

### Remarks on the authority of *Pteropus insularis*

“Hombron and Jacquinot, 1842” has long been the recognized authority for the name *Pteropus insularis* (e.g. Andersen 1912, Wilson and Reeder 1993, Flannery 1995), but a recent review of the early literature indicates this to be an error perpetuated over the years. The name was proposed in the results of a 19th century French expedition to Antarctica and Oceania known in abbreviated form as “*Voyage au Pole Sud*,” or in a longer and more complete form as “*Voyage au pôle sud et dans l’Océanie sur les corvettes l’Astrolabe et la Zélée exécuté par ordre du roi pendant les années 1837–1838–1839–1840 sous le commandement de M. Dumont-d’Urville*.” This work was published in seven parts (Histoire du Voyage, Zoologie, Botanique, Anthropologie, Géologie, Physique, and Hydrographie) in 23 volumes with six atlases. Many authors and editors contributed to the work and publication dates range from 1842–1854 for the different components. *Pteropus insularis* was described in Part II (Zoologie), volume 3, which was edited by Hombron and Jacquinot and divided into 3 sections under different authors. The first section, *Mammifères et Oiseaux* by H. Jacquinot and Pucheran (pp. 1–166), contains the description of *Pteropus insularis* on page 24. The two other sections are *Reptiles et Poissons* by H. Jacquinot and Guichenot (pp. 1–56) followed by *Crustacés* by H. Jacquinot and Lucas (pp. 1–107). Clark and Crosnier (2000) remark that the earliest verifiable date for volume 3 is 1854 (although 1853 appears on the title page), but Holthuis (2002: 419) pointed out that “as there is no evidence to prove that the volume was published after 1853…the date on the title page [see Holthuis 2002: figure 7] has to be accepted as correct.”

The description of *Pteropus insularis* by Jacquinot and Pucheran (1853) includes a reference to plate v, which was published in a separate *Atlas d’Histoire Naturelle, Zoologie*, edited by Hombron and Jacquinot. The plate is a composite of dorsal and ventral views of the body and dorsal, ventral, lateral, and frontal views of the skull of one of the two cotypes (skin 53 A, skull A. 6770) of *Pteropus insularis* in the Paris Museum (Andersen 1912). The plate is undated but the title page of the atlas bears the range of dates 1842–1853. However, a detailed study of the atlas by Clark and Crosnier (2000) revealed that the 40 plates were distributed in 28 livraisons, and that plate v was included in the 11^th^ livraison, the earliest verifiable date of which is 1844, according to British Library records. But the date of the plate is seemingly irrelevant as it identifies the species with a French vernacular name, *Rousette Insulaire*, not a scientific binomen. On the other hand, Jacquinot and Pucheran (1853: 24) give the name as “Rousette insulaire.—*Pteropus insularis* Homb. et Jacq.” Although Jacquinot and Pucheran (1853: 25) remarked “La Rousette insulaire a été trouvée par MM. Hombron et Jacquinot dans l’île d’Hogoleu [The island flying fox was found by MM. Hombron and Jacquinot in Chuuk.]”, and attributed authorship to them, there is no indication that Hombron contributed directly to the description. The authority of *Pteropus insularis* should revert to Jacquinot and Pucheran (1853). It is also noteworthy that Sherborn’s Index Animalium, which lists original sources of species names up to 1850, does not include *Pteropus insularis*.

### Distribution and status of *Pteropus phaeocephalus pelagicus*. Northern Mortlocks

Records of flying foxes from the northern Mortlocks are scanty, lacking in substantive detail, and confined to Losap Atoll; no records exist for Nama Island. Severance (1976) remarked that bats were present on Losap during his anthropological investigations in the late 1960s and early 1970s, but the information may have been based on hearsay from local residents as he did not later recall definitely seeing bats on the atoll (C. Severance, pers. comm.). Kachusy Silander, whom Severance first met on Pis Island, Losap Atoll, and who lived there until the early 1970s, stated in a telephone conversation (*fide* C. Severance, pers. comm.) that he never saw bats on the atoll, but recalled that the traditional chief at that time claimed that flying foxes lived on his parcel of land at the southeastern end of Pis. In 2004, DWB spoke with two former Losap Island residents who had relocated to Pohnpei. Tasiro Leg (pers. comm.) recalled occasionally seeing bats and throwing stones at them as a boy during the 1980s. Scaima Pamar (pers. comm.), age 78 in 2004, recalled seeing his mother and grandmother preparing bats to eat on Pis during the time of the Japanese administration in the 1930s or early 1940s. DWB also received a report from another former Losap resident, age 76 in 2004, who remembered a large group of bats arriving together at Losap shortly after World War II (*fide* Tridell Elitok, pers. comm.). They resided in a no longer present stand of mangroves and were caught and eaten by the islanders because of the scarcity of food at the time. H. Manner (pers. comm.), who spent one week collecting plants on Losap Island and adjacent islets in July 1988 (Manner and Sana 1995), did not remember seeing flying foxes then.

In the late 1980s, government officials on Weno, Chuuk Lagoon, reported bats present on Losap Atoll and said that a Losap islander was among several Mortlockese shipping bats to Weno for export to the Mariana Islands (Wiles 1992a), but whether any of the bats originated from Losap Atoll is unknown. Joakim Peter (pers. comm., College of Micronesia, Weno) informed DWB that the exporter from Losap resided on Weno at the time and worked mainly for his brother-in-law, who was from Ettal and also resided on Weno. J. Peter (pers. comm.) went on to say that the exporter from Losap may have been more involved with transportation and management operations than in collecting bats. Attempts to contact this exporter in 2004 were unsuccessful. Rainey and Pierson (1992), citing M. Henry and C. Glover in Chuuk Lagoon as sources, also reported bats from Losap but gave no additional details.

DWB saw no bats on Losap Atoll during a one-day visit that included 2–3 hrs each on Pis, Losap, and Lewel Islands in July 2004. Lewel, the largest island in the atoll, has extensive breadfruit-coconut forest typical of bat habitat elsewhere in the Mortlocks. It is used as a garden island by the people of Losap Island, but has no permanent settlement or resident families. He walked the length of Lewel through the center and returned via a shore route without seeing bats. None of the several Losap and Pis residents interviewed during his visit knew of bats occurring on the atoll.

DWB spent one week on nearby Nama Island without seeing any bats or encountering any resident islanders who had seen them. Mitaro Chosa (pers. comm.), deputy mayor of the island and age 58 in 2004, was firm in his conviction that bats had not occurred on Nama during his lifetime.

These few, and in some cases questionable, records together with the lack of sightings during this study suggest that flying foxes are absent from Nama Island, and are either recently extirpated or possibly still present in such small numbers as to be unknown to many islanders on Losap Atoll.

### Central Mortlocks

Girschner (1912) recorded flying foxes on Namoluk Atoll in about 1910, but gave no indication of their abundance. Bats were present on all of the atoll’s islands during the late 1960s and early 1970s and reportedly numbered “in the hundreds” (Marshall 1972: 17; 1975). During that time, bats were most common on the uninhabited islands of Amwes and Toinom (M. Marshall, pers. comm.).

DWB observed small numbers of bats on all the islands in July 2004. The encounter rate on Namoluk Island averaged four per hour during six 45-minute walks in the least disturbed parts of the coconut-breadfruit forest on four different days. Overall, an estimate of 150–200 bats was made for the atoll. However, Maikawa Setile (pers. comm.), deputy mayor of Namoluk, claimed that bats were more abundant at certain times, especially when breadfruit was in season. He recalled seeing large numbers of bats in the settlement earlier in 2004, with as many as 50 in one tree.

### Southern Mortlocks

There are few historical accounts of flying foxes in the southern Mortlocks. J. Nason (pers. comm.) reported “scores of fruit bats... possibly 100+” on Ettal Island, Ettal Atoll, during the late 1960s.

Wiles (1992b) reported bats on Ettal and Satawan Atolls being harvested for export in the late 1980s. A resident of Lukunor Atoll told us that he shot 50 bats on the atoll and another 150 bats on other southern Mortlock islands for this purpose during a 5-month period in 1986. These were sold to a Palauan man operating a trading boat, who provided the hunter with a .22 caliber air rifle and .410 gauge shotgun. The bats were steeped in boiling water (but not cooked), wrapped individually in plastic bags, and packed in ice prior to shipment.

During field surveys in 2004, DWB found bats to be uncommon on Ettal Atoll and estimated 75–100 to be present. He saw only five or six bats flying in and out of several breadfruit trees in the village on Ettal Island at sunset on 30 and 31 July, and observed only one bat during several walks elsewhere on the island and none on three of the atoll’s smaller outlying islands. One resident stated that he occasionally saw as many as 20–30 bats together in breadfruit trees in the settlement.

DWB judged bats to be common on Lekinioch Island, Lukunor Atoll, in 2004. He counted 53 bats in flight over the central taro patch and adjacent forest during 30 minutes near sunset on 2 August. No other station counts were made and no bats were seen during daytime walks among breadfruit and coconut trees in the settlement. However, the less disturbed areas of the island where bats were more likely roosting were not visited. DWB was unable to visit other islands in the atoll in 2004, but many of the people interviewed on Lekinioch, as well as former Lukunor residents living on Pohnpei at the time, reported bats on Oneop and many of the smaller islands. An overall estimate of 300–400 bats was made for the atoll.

In 2012, DWB visited all of the islands in Lukunor Atoll except Lekinioch. Small numbers of bats were recorded on eight of the 17 islands visited, as follows: Oneop, 5 bats; Piafa, 1; Kurum, 1; Pienemon, 4; Fanamau, 1; Sapull, 6; Sopotiw, 2; and Fanafeo, 1. Seven bats were noted flying between Sapull and Sopotiw Islands. No colonies were found and no population estimate was made for the atoll.

Surveys on Satawan Atoll in 2004 indicated that bats were common and regularly encountered, with an estimated 400–500 present. The largest numbers of bats observed during the study were on this atoll, with the greatest concentrations seen on Satawan and Ta, the two largest and southernmost islands in the Mortlocks. The encounter rate on Ta was eight per hour during walking surveys totaling 2.2 hours through breadfruit and coconut forest. The maximum number of bats observed during station counts was 81 in 25 min at Ta airport at sunset on 4 April. The largest aggregation observed during this study was 27 on Satawan Island (see Roosting below). One to 10 bats (usually less than five) were encountered per visit on other islands in the atoll, including Weito in the south; Kuttu, Orin, Pike, Mariong, Apisson, Lemasul, and Alengarik in the northwest; and Faupuker, Pononlap, and Fatikat in the east. Numbers of bats noted on Ta in 2012 matched or exceeded those recorded in 2004, and included many flying to or from Satawan Island.

### Natural history of *Pteropus phaeocephalus pelagicus*. Roosting

*Pteropus phaeocephalus pelagicus* characteristically roosted singly or in small, loose aggregations of 5–10 individuals, usually in the crowns of coconut and breadfruit trees in closed or nearly closed canopy forest outside of settlements. A maximum of 27 was seen together in the crowns of two adjacent coconut trees at the northern end of Satawan Island on 2 July 2004, with seven roosting along the rachis of a single palm frond and separated from each other by one or two body lengths. Bats were frequently observed hanging from tree branches and palm fronds, and occasionally clinging to the trunks of coconut trees a short distance below the crowns. Roosting bats were noted to occasionally stretch their wings and reposition themselves, or awake and relocate to another site. Some were seen crawling on the petiole bases of palm fronds and disappearing from view among the leaf axils.

### Diet

Ripe breadfruit is apparently one of the most preferred food items of *Pteropus phaeocephalus pelagicus*. DWB saw bats active in breadfruit trees at night on all the atolls having bats, and resident islanders invariably mentioned the fruit of breadfruit at the top of their lists of foods eaten by bats. Other foods reported by islanders were banana (*Musa* sp.; fruit), coconut (parts eaten unknown), papaya (*Carica papaya* L.; fruit), *Calophyllum inophyllum* L. (fruit), *Crataeva speciosa* Volkens (fruit), *Pandanus* cf. *tectorius* Parkinson (fruit), and *Ficus tinctoria* G. Forster (fruit). The only observation of bats feeding was on Weito Island, Satawan Atoll, at sunset on 3 July 2004, when three or four bats repeatedly flew in and out of the dense foliage of a pandanus tree. One flew away with a segment of ripe fruit and another dropped a segment while flying over the beach.

### Flight activity

**Figure 7.**
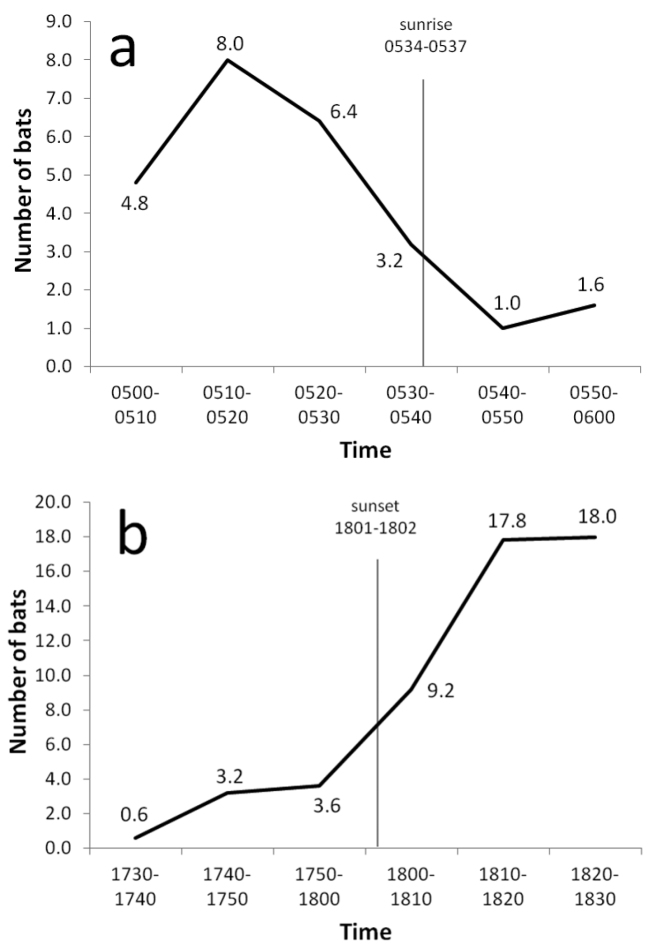
Mean number of bats observed per 10-minute count period during (**a**) five early morning counts from 23 June–6 July 2004 and (**b**) five evening counts from 22 June–3 August 2004 at the airport on Ta Island, Satawan Atoll, Mortlock Islands.

Flying foxes were observed flying at all hours, including midday, although some daytime activity was doubtless caused by the observer’s passage. Flight activity was greatest near sunset and sunrise (Figure 7) when bats moved between day roosts and foraging sites. Fewer bats were encountered at sunrise than at sunset, suggesting that some animals returned to roosts before daylight. The direction of evening flights probably was associated with the location of food resources, often ripe breadfruit according to local residents. During April and June 2004, with the exception of a few individuals settling in nearby trees, all bats observed during evening flights at the Ta airport station moved toward the western end of the island; in July and August, 19–40% of bats flew from west to east. Interisland flights within atolls occurred regularly near sunset, but none were recorded at other times (Table 5). No bats were seen flying between atolls.

**Table 5.** Numbers of bats observed flying between islands during six sunset and three sunrise counts at six different stations on Satawan Atoll, Mortlock Islands, 24 June–3 July 2004.

**Station^a^**	**Date**	**Time**	**Number of bats**	**Direction of flight**
1 Fatikat	26 June	1745–1815	0	
1 Fatikat	27 June	0515–0545	0	
2 Satawan (east end)	2 July	0500–0530	0	
2 Satawan (east end)	2 July	1730–1830	1	Fatikat to Satawan
2 Satawan (east end)	3 July	0430–0545	0	
3 Satawan (west end)	1 July	1725–1850	7	Ta to Satawan
4 Ta (east end)	25 June	1730–1830	0	
5 Ta (west end)	24 June	1715–1830	0	
6 Weito (east end)	3 July	1700–1835	8	Weito to Ta

^a^ See Figure 1 for locations.

### Reproduction

At least 50% of the bats observed on Namoluk Atoll in July 2004 appeared to be females with young. In some cases, young were evident only as a bulge beneath a female’s wing. Large volant young were occasionally seen in close proximity to or in body contact with their presumed mothers. Among five females collected on Namoluk Atoll on 21–22 July 2004, two contained single fetuses (crown-rump length 68 mm, mass 27 g, but with part of the brain case shot away; crown-rump length 42 mm) and a third had an immature male (forearm 78 mm, head-body length 110 mm) clinging to its ventral surface. Two females collected on Satawan Atoll in April 2003 each had a large young (head-body length 100, 100 mm; mass 62, 65 g) clinging to their ventral surface. Two adults were observed copulating in the crown of a coconut tree on Ta Island on 24 December 2002.

## Discussion

### Taxonomy

Our examinations and analyses of craniometric and pelage variation in the flying foxes of Chuuk State demonstrate minor but largely consistent morphological distinctions between flying foxes of the Mortlock Islands and those from the islands of Chuuk Lagoon and Namonuito Atoll. We regard these differences as indicating no more than subspecific distinction between these two regional groupings, which we refer to *Pteropus pelagicus* (*Pteropus phaeocephalus pelagicus* and *Pteropus phaeocephalus insularis*, respectively). A specimen from Namonuito Atoll, the enigmatic type series of *Pteropus laniger*, and a series of seized bats said to originate from Chuuk State, are all best referred to *Pteropus phaeocephalus insularis*. The closest relative of *Pteropus pelagicus* is the recently extinct *Pteropus tokudae* of Guam, which based on its consistently smaller size, shorter and narrower rostrum, darker coloration, and considerable geographic isolation from *Pteropus pelagicus*, is best regarded as a distinct species (cf. Tate 1934). An overlooked nineteenth century specimen in the Berlin Museum provides an indication that the islands of Chuuk Lagoon may have originally supported a second, larger species of flying fox (i.e., in addition to *Pteropus phaeocephalus insularis*), similar to *Pteropus pelewensis*, which may now be extinct in the archipelago. The possible historical extinction of this species, and those of *Pteropus tokudae* on Guam and *Pteropus pilosus* in Palau, highlights the vulnerability of remote Pacific *Pteropus* populations to insular extirpation and extinction, and the importance of further clarifying studies of the taxonomic status, ecology, and current conservation standing of flying fox populations from remote Pacific archipelagos (Wiles et al. 1997, Helgen et al. 2009).

The subspecific status of flying foxes in the northern Mortlocks remains unresolved. The nearest neighboring populations are comprised of *Pteropus phaeocephalus insularis* on the islands of Chuuk Lagoon 66 km to the northwest and *Pteropus phaeocephalus pelagicus* on Namoluk Atoll 110 km to the south. Both distances are perhaps within the flight capabilities of the species. However, based on its nearer distance and much larger land area that would support a larger bat population producing potentially more dispersing individuals, Chuuk Lagoon would appear to be a more likely source of animals colonizing Losap Atoll and Nama Island. Interisland movements of up to 119 km and corresponding genetic exchange have been reported in *Pteropus mariannus* in the Mariana Islands (Wiles and Glass 1990, Brown et al. 2011), but this species is substantially larger (forearm 134–154 mm), and perhaps a more powerful flier, than *Pteropus pelagicus*.

### Status and natural history

The entire population of *Pteropus phaeocephalus pelagicus* is probably currently restricted to the four atolls comprising the southern and central Mortlock Islands, which total 10.1 km^2^ in size. A few additional bats may be present on Losap Atoll in the northern Mortlocks, but it is unclear which subspecies these might represent. Our study provides the first population estimate for *Pteropus phaeocephalus pelagicus*, with approximately925–1,200 bats present in 2004. About 75% of the population occurs on the two atolls with the largest land areas: Satawan and Lukunor. Resident islanders in the southern and central Mortlocks reported that bats were more common in the past, but that numbers appeared to have remained relatively stable in recent years. Residents also stated that bat abundance seems to fluctuate with the seasonal availability of food, especially breadfruit, but this impression likely comes from the bats’ strong attraction to villages during the peak fruiting season of breadfruit.

Some double-counting of flying foxes may have occurred during our survey due to movements of bats between atolls. However, this problem was probably minor because our primary survey period when all six island groups in the Mortlocks were visited was limited to a 6-week period from 22 June to 5 August 2004, thus reducing the likelihood of significant inter-atoll flights.

Recent data are lacking on the status of *Pteropus phaeocephalus insularis*. The main population in Chuuk Lagoon probably numbered in the mid-thousands in the early 1980s, but was reduced in abundance by commercialized hunting in the latter part of that decade (Wiles 1992b, 1992c). The only records from Namonuito Atoll are two specimens (BMNH 15.1.18.1, 15.1.18.2) collected in 1914. DWB did not sight any bats while visiting Onoun (Ulul) Island in this atoll on 3–7 January 2003, and none of the local residents queried reported seeing bats. However, no information was obtained for the islands on the eastern and northern sides of the atoll.

Our survey efforts, including conversations with islanders, failed to confirm the presence of two other bat species in the Mortlock Islands, both of which are based on questionable early records. Thomas (1882) and Andersen (1912) listed *Pteropus molossinus* Temminck (originally reported under the name *Pteropus breviceps*; see Thomas 1887) as present in the Mortlocks, based on a single specimen collected by Kubary that supposedly originated from the island group. *Pteropus molossinus* is otherwise recorded only from Pohnpei and nearby Ant and Pakin Atolls (Wiles 1992d, Buden 1996a, 1996b). Our findings support Rainey and Pierson’s (1992) belief that this record is erroneous, though it is also possible that *Pteropus molossinus* was more widespread in the recent past and is now extinct or extremely rare in the Mortlocks. We also did not detect the insectivorous emballonurid *Emballonura semicaudata sulcata* Miller, which is firmly recorded only from Chuuk Lagoon’s main islands and Pohnpei (Koopman 1997). Schmeltz and Krause (1881: 298), in listing the mammals that Kubary recorded in the Mortlocks, remarked about “…noch einen von Ruk her eingefuhrten Hund, von wo auch ab und an eine *Emballonura* hierher verweht wird […a dog imported from Chuuk and sometimes an *Emballonura* blown in from that same place].” Further along in the same text, and in comparing the fauna of Nukuoro Atoll, Pohnpei State, with that of the Mortlocks, Schmeltz and Krause (1881: 331) stated, with respect to Nukuoro, “…von Säugetieren kommt nur die Ratte vor; *Pteropus* und *Emballonura* fehlen […of the mammals, there is only the rat; *Pteropus* and *Emballonura* missing.].” These unconfirmed allusions to *Emballonura semicaudata* in the Mortlocks are probably best treated as hypothetical or indicative of occasional vagrant occurrence.

Atoll-dwelling populations of *Pteropus* are of ecological interest because of their occurrence on islands with impoverished and often highly human-altered floras (see Mueller-Dombois and Fosberg 1998). Our study of *Pteropus phaeocephalus pelagicus* supports the findings of others (Dolbeer et al. 1988, Wiles et al. 1991, Holmes et al. 1994) that although flying fox diets appear to comprise relatively few plant species on atolls, these islands nevertheless can support sizeable population densities approaching or exceeding 100 bats per km^2^. The fact that atoll habitats have experienced widespread conversion to coconut-dominated forest and that a number of cultivated fruit-producing tree species has been introduced may enhance the productivity of these islands for flying foxes (Wiles and Brooke 2009).

Most species of *Pteropus* are seasonal breeders with births synchronized over a period of several months (O’Brien 1993). Contrary to this pattern, our limited observations suggest that births in *Pteropus phaeocephalus pelagicus* occur over a longer time frame and that females with dependent young are present for at least 8 months of the year. Two other Micronesian taxa, *Pteropus yapensis* in Yap and *Pteropus mariannus* on Guam, are among the few species of flying fox known to breed continuously (Wiles 1987b, Falanruw 1988).

### Threats to populations of *Pteropus phaeocephalus pelagicus*

With a small population size and a geographic range comprised of many small low-lying islands totaling <12 km^2^, flying fox populations in the Mortlock Islands are highly vulnerable to environmental changes and certain human activities. The apparent depletion or possible extirpation of bats on Losap Atoll in the northern Mortlocks during the past 60 years underscores this vulnerability.

Sea level rise associated with climate change may represent the greatest long-term threat to *Pteropus phaeocephalus pelagicus* and other populations of atoll-dwelling *Pteropus* (Rainey 1998). One of the most recent climate models forecasts a rise in sea level of 0.57–1.10 m by 2100 and 1.84–5.39 m by 2500, although much uncertainty exists over the latter estimate (Jevrejeva et al. 2012). Rises of this magnitude certainly have the potential to submerge atolls throughout the world or greatly reduce atoll land areas and change vegetation patterns, and therefore put *Pteropus phaeocephalus pelagicus* at considerable risk of extinction within this or the next several centuries. Lukunor Atoll in the Mortlocks and some other Micronesian atolls experienced several extreme high tides between 2007 and 2009 that flooded islets for several hours per event (Fletcher and Richmond 2010, Keim 2010). At Lukunor Atoll, the resulting salinated soil from the initial flooding in 2007 damaged or killed 78% and 55% of the breadfruit trees on Oneop and Lekinioch islands, respectively (Keim 2010), substantially reducing an important food for *Pteropus phaeocephalus pelagicus* on the atoll. Despite this, some experts believe that some atoll islands may be more resilient to sea level rise than previously thought and may be able to maintain their land area through continued sediment accretion as long as geomorphic processes are not substantially altered in the future by ocean warming and acidification, changes in storm occurrence, and reductions in atoll vegetation (Woodroffe 2008, Webb and Kench 2010). However, even if atoll islands persist in the future, it is unknown what type of vegetation may remain and whether it could support populations of flying foxes.

The negative impacts of severe cyclonic storms on *Pteropus* populations are well documented and occasionally include significant population declines (e.g., Cheke and Dahl 1981, Pierson et al. 1996, McConkey et al. 2004, Esselstyn et al. 2006, Wiles and Brooke 2009). The vast majority of bat mortalities occur in the aftermath of storms following depletion of food resources, loss of protected roosting sites, and in some cases increased post-storm hunting pressure. The Mortlock Islands are positioned along the southern edge of the typhoon belt present in the western North Pacific Ocean. Records since the early twentieth century suggest that individual islands in the Mortlocks experience devastating typhoons and massive damage to plant communities two to four times per century (Nason 1970, Marshall 1975, 1976, Joint Typhoon Warning Center 1980–2011, Reafsnyder 1984). However, even the largest storms rarely inflict serious habitat damage across the entire archipelago (e.g., Marshall 1979) and typically leave flying foxes and other wildlife with habitat refugia on some islands.

In 1976, Typhoon Pamela killed most of the tree crops (e.g., breadfruit, coconut, and banana trees) on the islands of Ettal, Namoluk, Kuttu, Oneop, Moch, and Nama (Marshall 1976, 1979), including 95% of the breadfruit on Kuttu (Reafsnyder 1984) and all breadfruit, coconuts, and bananas on Ettal, where breadfruit trunks and limbs were stripped bare (M. Marshall, *fide* J.Nason, pers. comm.). In contrast, the islands of Losap, Ta, Satawan, Pis, and Lukunor experienced much lower losses of these trees. Ettal, Namoluk, and Kuttu were also entirely submerged by high sea surge for 15–18 hrs during this typhoon (Marshall 1979). During Typhoon Phyllis in 1958, nearly 75% of all trees at Namoluk Atoll were fully uprooted with the remaining trees being mere stumps 5–7 m tall (Davis 1959, in Marshall 1975).

Current model projections suggest that typhoon intensity will increase, but frequency may decrease, in the western Pacific as climate change progresses over the next century (Knutson et al. 2010). Typhoon impacts on atolls may be exacerbated when combined with projected rises in sea level.

The extent that Mortlock bat populations have been affected by severe typhoons in the past is undocumented. However, Marshall (1976) remarked that although few birds were encountered on the hardest hit islands 6–10 days after Typhoon Pamela, flying foxes appeared to survive the storm fairly well, with good numbers seen flying about searching in vain for food and shaded roosting sites. *Pteropus phaeocephalus pelagicus* has doubtless had a long history of exposure to intense typhoons and has been able to adjust to these periodic environmental disruptions and presumed population reductions, possibly via recruitment from surrounding islands or simply by population renewal by survivors.

Centuries of human occupation have greatly altered the vegetation of the Mortlocks, but anthropogenic disturbance is now low despite the atoll’s high human population density. The coconut-breadfruit-pandanus forests where bats roost and feed are economically important to islanders, and they manage this resource sustainably. Cutting and clearing of the undergrowth occurs sporadically and is usually done on small, family-owned plots, but widespread cutting of forest does not occur. Additionally, a high emigration rate of Mortlockese to the larger islands of Micronesia and to overseas locations for better job opportunities (Marshall 2004) has reduced human population growth and stress on the environment.

Overhunting has contributed to declines in flying fox populations in many areas of the Indo-Pacific region (e.g., Wiles et al. 1989, 1997, Craig et al. 1994, Brooke and Tschapka 2002, Lee et al. 2005, Wiles and Brooke 2009, Harrison et al. 2011) and may have done so in the Mortlocks in the past. Between 1986 and 1989, large numbers of *Pteropus pelagicus* were harvested in Chuuk State and exported to meet the demands of the commercial markets for bats on Guam (5,108 bats; Wiles 1992b) and in the Commonwealth of the Northern Mariana Islands (229 bats; Stinson et al. 1992). An unknown, but possibly sizable, number of these bats originated from the Mortlocks (Rainey 1990, Wiles 1992a, 1992b). Some of the bat exporters and/or hunters involved in this trade were from Losap, Ettal, Satawan, and Lukunor Atolls (Wiles 1992a, this study). The impacts of this harvest on bat populations in the Mortlocks were never quantified. The listing of all Micronesian species of *Pteropus* on Appendix I of the Convention on Trade in Endangered Species of Wild Flora and Fauna (CITES) in 1989 halted, or at least greatly reduced, commercial hunting of bats in the Federated States of Micronesia for export to the Marianas (Wiles 1992b, Wiles and Brooke 2009). It is uncertain to what extent illegal shipments of bats from Chuuk State may have occurred since then, but the amount is probably insignificant.

Like the vast majority of islanders from Chuuk, Pohnpei, and Kosrae states, Mortlockese almost universally disdain flying foxes as food. From the late 1960s to 1980, anthropological researchers noted that bats were not hunted or eaten on Ettal Island (Nason 1970), Namoluk Atoll (Marshall 1972), Losap Atoll (Severance 1976), Lukunor Atoll (Borthwick 1977), and Kuttu Island, Satawan Atoll (C. Reafsnyder, pers. comm.). Marshall (1972) remarked that mention of consuming bats, as done by residents of the Marianas, brought expressions of revulsion to Namoluk islanders. He also noted that bats were disliked in part because of their habit of urinating on themselves while roosting and their depredation of breadfruit, papayas, and bananas. J. Nason (pers. comm.) stated that in the Mortlockese ethnotaxonomy, bats were essentially considered as rats with wings and were never eaten. Paraphrasing a folktale told to DWB by M. Setile of Namoluk Atoll,

*Once, long ago, the rat was envious of the bat’s wings. The rat asked the bat if he could borrow his wings for a short time just to have a brief experience of flying; he promised to return them soon. The bat allowed the rat to use his wings. But the rat lied and flew away with no intention of returning the wings. Now, what was rat is bat and the original bat is the rat*.

Flying foxes may have been a part of the Mortlockese diet in the past, when reliance on local foods was greater and food supplies from Chuuk Lagoon and Pohnpei were not so readily available, and bats may still be utilized on occasion especially when other foods become scarce, such as after typhoons. Girschner (1912) included bats among the foods eaten by Namoluk islanders, and elders from Pis and Losap Islands told DWB of bats being eaten during the 1930s or 1940s. During our visits to the Mortlocks, no evidence was observed of bats being hunted, eaten, or killed to limit crop damage. Conversations with resident islanders throughout the central and southern Mortlocks indicated that local consumption of bats was apparently confined to a few individuals, who ate them from time to time, and to occasional visitors (largely friends and relatives) who acquired a taste for bats elsewhere.

Potential predators of flying foxes in the Mortlocks include four non-native species: cats (*Felis catus* Linnaeus), rats (*Rattus exulans* (Peale) and *Rattus rattus* complex; Wallace et al. 1972), and Pacific monitor lizards (*Varanus indicus* (Daudin)). Cats and rats are widely distributed among all major island groups, though not necessarily on every island. Although both may prey opportunistically on bats, Rainey (1998) noted that bats roosting in tree canopies have managed to coexist with these predators in most settings. At Namoluk Atoll, M. Setile (pers. comm.) once observed cats on the roof of a cooking shed keeping close watch on bats feeding on fruits of an adjacent *Crataeva speciosa* tree, but the bats kept a safe distance away. On another occasion, he saw a cat carrying the wing of a bat, but did not know how it was acquired. Monitor lizards are localized in distribution, occurring on only a few islands in the southern Mortlocks, including Lekinioch and Satawan islands (Buden 2007), where bat populations are large relative to elsewhere in the Mortlocks. Monitor lizards are opportunistic predators that consume a variety of invertebrate and smaller vertebrate prey, and are effective climbers. Flying foxes have not been reported in their diet (Dryden 1965, Losos and Greene 1988, McCoid and Witteman 1993, Dryden and Ziegler 2004, Philipp et al. 2007), but monitor lizards have been observed climbing in trees and approaching roosting *Pteropus mariannus* on Guam (D. Janeke, pers. comm.).

### Specimens examined

***Pteropus pelagicus pelagicus***

Mortlock Islands: Namoluk Island, Namoluk Atoll (4 skins and skulls and 2 fluid preserved adults, field numbers 12–17, plus two unnumbered fetuses and one unnumbered young, all COM collections); Satawan Island, Satawan Atoll (11 skins and 9 skulls, COM field numbers 1–11); “Mortlock Islands” (in fluid with skull extracted, BMNH 82.10.27.4 [holotype of *phaeocephalus*]).

***Pteropus pelagicus insularis***

Chuuk Lagoon islands and Namonuito Atoll: “Ruck” (skull, MNHN 1996-2112 (apparently originally A6770 *fide* Andersen 1912) [lectotype of *insularis*]; 2 skulls, ZMB 5698 and an unnumbered specimen collected in 1907; skull extracted from fluid specimen, BMNH 98.1.2.1); Uala (= Weno) (4 skins and skulls, USNM 151563–151566); Moen (= Weno) (4 skins and skulls, BYUH 248, 249, 253, 254); Namonuito (skull, BMNH 15.1.18.1); “Truk” (1 skin and skull plus 13 skulls [confiscated in Guam, probably all from Chuuk Lagoon, but possibly also outlying islands; all tentatively identified as *Pteropus phaeocephalus insularis*], AMNH 249954–249969); “Samoa” (emended to Caroline Islands [probably Chuuk Lagoon islands]) (skin and skull, USNM 37815 [lectotype of *laniger*], and skin, MCZ 7023); no locality (ANSP 6196, skull).

***Pteropus tokudae***

Guam (2 skins and skulls, AMNH 87117 [holotype of *tokudae*] and 87118, and 1 skull, AMNH 256558).

***Pteropus* cf. *pelewensis***

“Ruck” (skull, ZMB 5697); Pohnpei (skull, ZMB A4065).
